# Integrative Physiological Strategies for Monitoring Demands in Functional Fitness

**DOI:** 10.3390/sports13110381

**Published:** 2025-11-04

**Authors:** Manoel Rios, David B. Pyne

**Affiliations:** 1 Piaget Research Center for Ecological Human Development, Higher School of Sport and Education, Jean Piaget Polytechnic Institute of the North, 4405-678 Vila Nova de Gaia, Portugal; 2 Centre of Research, Education, Innovation and Intervention in Sport and Porto Biomechanics Laboratory, Faculty of Sport, University of Porto, 4200-450 Porto, Portugal; 3 Research Institute for Sport & Exercise, University of Canberra, Canberra 2617, Australia; david.pyne@canberra.edu.au

**Keywords:** monitoring, testing, assessment, wearable technology, framework

## Abstract

An integrated physiological model would be useful for monitoring internal load in functional fitness, including formats like CrossFit and Hyrox. Traditional performance metrics often neglect internal strain, energy system engagement, and neuromuscular fatigue, central to these modalities. Oxygen uptake kinetics, metabolic profiling, heart rate and heart rate variability monitoring, and neuromuscular fatigue assessment can be employed for load monitoring. Breath-by-breath oxygen uptake analysis characterizes aerobic activation and recovery. Metabolic stress is estimated via indirect calorimetry and capillary blood lactate to quantify oxidative, glycolytic, and phosphagen contributions. Heart rate is tracked continuously to assess session intensity, while heart rate variability provides insights into autonomic recovery. Neuromuscular fatigue can be assessed via countermovement jump performance, offering sensitive measures of recovery and training tolerance. Portable tools such as the Cosmed K5**™**, Lactate Pro 2, heart rate sensors, and force platforms support real-time monitoring in training and competitions. Rather than advocating for the continuous use of advanced tools, the model promotes strategic integration of high-precision methods for research, and practical, low-cost alternatives (e.g., heart rate monitoring, session rating of perceived exertion, or jump analysis apps) for day-to-day coaching. This approach enables early detection of maladaptation, supports individualized training adjustments, and improves safety and performance outcomes. Ultimately, this framework bridges physiological science and real-world practice, providing value across both applied and research settings.

## 1. Introduction

Functional fitness has emerged over the past two decades as one of the most prominent trends in both competitive and recreational exercise [1,2,3]. This style of training integrates elements of weightlifting, gymnastics, and endurance sports into a single session, often performed at maximal or near-maximal intensity and designed to enhance a broad spectrum of physical capacities [1,2,4]. The appeal of functional fitness lies in its variety, efficiency, and potential to simultaneously stimulate adaptations in strength, cardiovascular endurance, and metabolic conditioning [2,4,5]. As a result, functional fitness training has gained popularity among recreational exercisers, elite sport and tactical populations, such as military personnel and first responders [1,6].

CrossFit and Hyrox have emerged as two globally prominent functional fitness formats. CrossFit is characterized by the absence of a fixed structure, relying instead on daily training sessions known as the “workout of the day” [5,6,7,8].These sessions are purposefully designed to simultaneously challenge multiple energy systems by combining high movement density, technical variability, and substantial metabolic demand [5,6]. To evaluate fitness and monitor changes in work capacity, CrossFit incorporates standardized benchmark workouts, with Cindy and Fran being among the most extensively studied in the literature [9,10,11,12]. These benchmark sessions follow consistent structures and conditions, allowing for reliable performance comparisons across athletes and training cycles [5,6]. Evidence from recent investigations indicates that these workouts impose substantial cardiovascular and neuromuscular demands, including elevated blood lactate concentrations, rapid increases in oxygen uptake, and significant decrements in muscle function following exercise [9,10,11,13,14]. These physiological responses are modulated by factors such as workout structure, athlete training status, and sex, making individualized monitoring and training load regulation an ongoing challenge in functional training [6,13,15].

Unlike CrossFit, Hyrox competitions follow a fixed and predictable structure comprising eight standardized exercises (ski ergometer, sled push, sled pull, burpee broad jump, rowing ergometer, farmers carry, sandbag lunges, and wall balls), interspersed with eight 1000-m runs [16,17]. The event always begins with a running segment, followed by alternating bouts of running and exercise stations in a consistent sequence. Exercise loads and distances are systematically adjusted according to sex, competition division, and format (e.g., Individual Open, Individual Pro, Doubles, and Relay), enabling participation across a wide range of fitness levels [16,17]. Recent research has demonstrated that Hyrox imposes substantial physiological stress, with participants completing the competition in ~86 min, predominantly at intensities classified as “hard” to “very hard” (i.e., >80% of maximal heart rate) [16]. Perceived exertion ratings and blood lactate concentrations also indicate substantial anaerobic involvement, especially during the heavier resistance-based stations [16]. However, performance appears to be more strongly associated with aerobic capacity, endurance training volume, and body composition (highlighting a greater reliance on cardiorespiratory endurance and muscular stamina rather than maximal strength) [7,8,16,17]. This physiological profile distinguishes Hyrox from other functional fitness modalities such as CrossFit (particularly in terms of exercise type, external load, volume, and recovery), indicating that metabolic efficiency, pacing strategies, and the ability to transition between high-load tasks and sustained running efforts are key determinants of success in this format [16].

CrossFit and Hyrox are prominent examples of physiologically demanding modalities that challenge multiple bodily systems under highly variable training conditions. Despite their rapid growth and widespread adoption, there remains a lack of specific and validated methods to effectively monitor and adjust training loads within these contexts. Established parameters from traditional sports (e.g., oxygen uptake kinetics, lactate threshold assessments, and neuromuscular fatigue tests) have yet to be fully adapted to the complex demands of functional fitness. To address this gap, we propose a structured three-step model: (i) identification and evaluation of physiological tools most appropriate for functional fitness environments; (ii) implementation of applied studies with both athletes and coaches to test and refine these tools in real-world settings; and (iii) translation of these findings into clear, actionable guidelines that provide evidence-based application of physiological data.

Rather than proposing a novel theoretical construct, this model is designed as a practical framework to guide applied monitoring in functional fitness environments. Its originality lies in integrating established physiological methods (e.g., oxygen uptake kinetics, lactate profiling, and neuromuscular fatigue monitoring), into a structured approach that supports both scientific investigation and daily coaching practices [10,18]. While advanced metrics such as breath-by-breath oxygen uptake analysis and lactate sampling may be more applicable in research contexts to deepen physiological understanding, more accessible tools (e.g., heart rate monitors, session rating of perceived exertion, and mobile apps for jump assessment), offer practical value for athletes and coaches [13,19,20]. By combining these methods strategically, the model enhances early detection of maladaptive responses, supports individualized adjustments, and promotes effective load management across training cycles.

## 2. An Integrated Physiological Model

This section proposes an integrated framework that combines cardiorespiratory dynamics, metabolic system profiling, and neuromuscular assessment to quantify training stress and guide evidence-based decision-making.

### 2.1. Cardiorespiratory Function

Quantifying the dynamic features of oxygen uptake kinetics has gained popularity in exercise physiology as a means of identifying the mechanisms underlying the control of muscle oxygen consumption during exercise [21]. Breath-by-breath analysis of oxygen uptake has emerged as a valuable, non-invasive method to characterize the dynamic behavior of the aerobic energy system during exercise [21,22]. Key parameters (e.g., amplitude, time delay, and time constant) provide critical insights into the speed and efficiency of the oxidative response to sudden workload transitions [21,22]. This approach has been extensively validated in cyclic sports such as cycling, running, swimming, and rowing, where oxygen uptake kinetics serve as sensitive markers of aerobic fitness and fatigue resistance [22]. Recent studies have demonstrated that oxidative metabolism can contribute substantially even during short, high-intensity sprint efforts [23]. For instance, in 200-m cycling sprints, the aerobic contribution may exceed 40%, depending on the duration of the event and the recovery dynamics involved [24]. This is especially relevant in modalities like CrossFit^®^ or Hyrox, which involve non-steady-state conditions and frequent transitions between exercises that demand distinct motor and metabolic profiles.

For instance, during the Fran benchmark workout, oxygen uptake kinetics exhibited an amplitude of ~36 mL·kg^−1^·min^−1^, a time constant near 24 s, and heart rate peak values close to 185 bpm, indicating rapid activation of the oxidative system consistent with the severe intensity domain [10]. Similarly, during the Isabel benchmark workout (~120 s), oxygen uptake kinetics displayed a prominent fast component without sufficient time for a slow component to emerge. The high contraction velocity was associated with marked cardiorespiratory (peak oxygen uptake ~47 mL·kg^−1^·min^−1^ and heart rate peak ~178 bpm) and metabolic stress (blood lactate ~21 mmol·L^−1^), ultimately influencing recovery trajectories [23]. Integrating such kinetics into training monitoring enables more precise evaluation of aerobic system engagement and recovery. During a simulated Hyrox competition, peak heart rate reached 185 bpm and blood lactate peaked at 8.5 mmol·L^−1^, with significantly higher values recorded during exercise stations compared to running bouts [16].

These acute cardiorespiratory and metabolic responses can be further contextualized by assessing autonomic nervous system modulation through heart rate variability, a sensitive and complementary indicator of internal load and recovery status [25,26]. heart rate variability is a non-invasive marker commonly used to monitor autonomic nervous system activity, particularly parasympathetic modulation. The root mean square of successive differences (RMSSD) and its natural logarithm (LnRMSSD) are frequently applied to assess internal training load, fatigue, and recovery [27]. Functional fitness sessions can acutely reduce heart rate variability, indicating increased sympathetic activation. In one study, after benchmark CrossFit^®^ workouts (e.g., Fran, Megan, and Diane), RMSSD values reduced sharply during exercise (Fran: 49.8–12.0 ms; Megan: 49.8–5.5 ms; Diane: 54.7–4.4 ms), with only partial recovery observed after 60 min (Fran: 27.5 ms; Megan: 22.1 ms; Diane: 29.1 ms) [27]. Heart rate variability-guided programming can reduce the frequency of high-intensity sessions without compromising performance outcomes, supporting its use in individualized training management [25,26].

### 2.2. Bioenergetic Profiling

In addition to oxidative dynamics, quantifying the relative contributions of the phosphagen, glycolytic, and oxidative energy systems provides a more comprehensive understanding of the energetic demands elicited by functional fitness. This bioenergetic profiling enables practitioners to align training loads with the targeted energy pathways, ultimately enhancing metabolic specificity and adaptive outcomes [28]. The most widely accepted method for estimating system-specific energy contributions in applied settings involves the combined use of indirect calorimetry and blood lactate measurements [28]. The oxidative contribution is calculated by integrating the area under the oxygen uptake curve above resting baseline during exercise, and converting it into energy using a caloric equivalent of 20.9 kJ·L^−1^ [10,23]. The glycolytic system is typically estimated based on the net increase in blood lactate post-exercise, assuming a standard energetic equivalent of 3 mL·kg^−1^·mM^−1^ [10,23]. A comparative analysis across four cyclic modalities showed that running elicited the highest oxidative contribution (~80%) and the lowest glycolytic (~12%), followed by rowing (74% oxidative, 16% glycolytic) and cycling (74% oxidative, 15% glycolytic), while swimming presented the lowest oxidative (~73%) and highest glycolytic (~12%) contributions [22]. In recent investigations applying this approach to benchmark workouts, Fran exhibited an energy profile comprising ~62% oxidative and ~31% glycolytic contribution [10], while Isabel demonstrated about 40% oxidative and 45% glycolytic involvement [23]. These differences highlight the need to account for modality-specific metabolic profiles when assessing energy system engagement in functional fitness.

The phosphagen system, which is often underrepresented in field assessments, can be estimated through two validated non-invasive strategies [28,29]. The first involves mono-exponential modeling of the rapid phase of post-exercise oxygen uptake, interpreted as the oxygen cost associated with the resynthesis of adenosine triphosphate and phosphocreatine [28,29]. To improve the accuracy of this method, breath-by-breath data are typically edited to remove errant breaths (usually caused by swallowing, coughing, or signal artifacts), following established procedures [29]. Peak oxygen uptake values are then obtained by backward extrapolation from the first 20 s of recovery, excluding the cardiodynamic phase, and smoothed using a moving average approach [29]. An energetic equivalent of 20.9 kJ·L^−1^ is applied to quantify the total phosphagen contribution [22,29]. Another approach considers a known decline in creatine phosphate concentration within active muscles, based on kinetic data from the transition between rest and exhaustion [22,29].

In a recent study, we compared two methods of assessing the phosphagen energy contribution [29]. No significant differences were observed between the results (31 vs. 30 kJ for the oxygen uptake recovery-based and creatine phosphate depletion-based methods, respectively). Although there are certain methodological limitations, such as the potential delay in the onset of oxygen uptake recovery and use of predetermined creatine phosphate values per kg of wet muscle mass, the outcomes indicate that both methods yield comparable estimates of phosphagen energy contribution [29]. Furthermore, a key advantage of these approaches is their non-invasive nature, which clearly distinguishes them from muscle biopsy techniques [28].

### 2.3. Neuromuscular Fatigue

Neuromuscular fatigue also represents a key component of internal load monitoring and reflects the transient reduction in the muscle’s ability to generate force or power following intense physical exertion [9,10]. One of the most validated and practical field-based tools for assessing this fatigue is the countermovement jump, which offers sensitive and non-invasive insights into neuromuscular status when administered before and after training sessions [9,10,30]. Meaningful decrements in countermovement jump performance (e.g., ~8% in jump height, ~6% in peak force and ~4% in maximum velocity), have been reported following benchmark workouts like Fran, and are considered indicative of residual fatigue and reduced neuromuscular output [10]. A direct comparison of distinct CrossFit workouts showed that countermovement jump performance declined substantially after the workout Cindy and the Olympic lifting workout, with reductions of ~7% and ~8% in jump height, and ~14% and ~3% in maximum velocity, respectively [9].

Countermovement jump assessments can provide actionable information to guide recovery strategies, adjust training loads, and identify early signs of non-functional overreaching [30]. This field-based diagnostic approach is supported by evidence demonstrating its sensitivity to detect meaningful fatigue responses in sport-specific training environments [30,31]. Neuromuscular fatigue diagnostics also provide actionable insights into athlete readiness and recovery status [9,10]. Moreover, longitudinal tracking of internal load indicators (when integrated with external training metrics such as total volume and session density) enhances the capacity to detect maladaptive patterns or early signs of non-functional overreaching [9,30]. These insights are particularly valuable in high-frequency training settings common to functional fitness disciplines, allowing for more individualized and adaptive periodization strategies [31].

**Figure 1:**
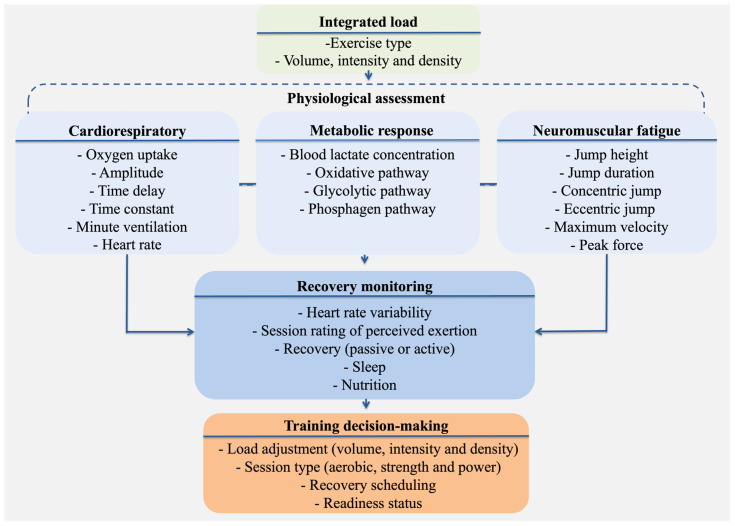
Conceptual model illustrating the integration of real-time and post-exercise physiological monitoring tools for internal load assessment in functional fitness.

Integration of real-time and post-exercise physiological metrics (spanning cardiorespiratory responses, metabolic stress, and neuromuscular fatigue), enables a comprehensive evaluation of internal load in functional fitness. This multidimensional approach supports the interpretation of acute responses (e.g., oxygen uptake kinetics, blood lactate, and countermovement jump metrics) in conjunction with recovery markers (e.g., sleep, nutrition, and heart rate variability), informing individualized load regulation. For example, a delayed oxidative response paired with elevated lactate and suppressed jump performance may reflect insufficient recovery or the onset of non-functional overreaching. Figure 1 presents the conceptual model guiding this integrated monitoring framework, linking physiological domains to training.

## 3. Integrating Technology and Physiology

Advances in portable technology enable integration of physiological assessments into training and competition environments, equipping coaches and sports scientists with tools to monitor internal load in real time [32,33]. Wearable oxygen uptake analyzers, handheld lactate monitors, heart rate sensors, and compact force platforms collectively enhance the ability to assess physiological responses during functional fitness sessions, with ecological validity and minimal interference with movement execution [32,33]. Integration of these tools into the athlete monitoring process allows for more precise load management, identification of recovery needs, and customization of training programs based on individual responses and adaptation trajectories [32,33].

A notable advancement is the Cosmed K5™, a portable breath-by-breath metabolic system that measures oxygen uptake, carbon dioxide output, and ventilatory variables during unrestricted movement [10,33]. Peak oxygen uptake and related parameters are typically derived from the final 30–60 s of exercise, with raw data filtered to exclude artifacts (e.g., coughing, and signal loss), retaining only values within ±3 standard deviations and applying smoothing (e.g., 3-breath or 10-s moving averages) [10,34]. To extract oxygen uptake kinetics, data are modeled using mono- or bi-exponential curve fits, with VO_2_FITTING software playing a key role in this process [34]. This platform enables precise estimation of kinetic parameters (e.g., amplitude, time delay, and time constant), while supporting bootstrapping (e.g., 1000 resamples) to compute confidence intervals and allowing for exclusion of the cardiodynamic phase [34]. VO_2_FITTING integrated signal smoothing and model diagnostics make it a robust, practical choice for analyzing oxidative system dynamics in the field.

Lactate monitoring has become increasingly accessible in field settings through portable devices such as the Lactate Pro 2™ that provide reliable results from small capillary blood samples (5 μL) within 15 s [9,10,23]. For accurate assessment, samples are typically collected from the fingertip or earlobe at standardized intervals (commonly at baseline and at 1, 3, 5, and 7 min post-exercise), to capture the lactate peak and clearance kinetics [10,23]. Although practical, portable lactate devices can be sensitive to variability in peak measurements and monitoring requires strict adherence to standardized sampling protocols.

Heart rate monitoring remains a fundamental parameter in internal load assessment, particularly during functional fitness [9,13]. Tools such as the Polar H10 and Garmin HRM-Pro offer high-resolution, artifact-resistant data even under dynamic movement patterns and are compatible with wireless transmission and cloud-based analysis platforms [25,32]. More recently, heart rate variability monitoring via validated smartphone applications (e.g., HRV4Training and Elite HRV) has emerged as a practical tool for tracking autonomic recovery [35,36]. These apps typically measure the LnRMSSD, a time-domain index that reflects parasympathetic activity [25]. Data collection is usually performed under standardized conditions, such as upon waking and in a seated or supine position, to ensure consistency and reduce external variability [37]. Chest strap monitors, including the Polar H10, are typically employed to obtain ECG-grade signal quality, even in unsupervised or field-based environments [36]. The raw R-R interval data are processed to remove ectopic beats and artifacts, using either automated algorithms or manual inspection, with commonly applied filtering thresholds ranging between 250 and 1200 milliseconds [35,36]. Repeated measurements are interpreted relative to individual baselines, typically through rolling averages (e.g., 7-day mean) or smallest worthwhile change thresholds, which facilitates the detection of meaningful physiological fluctuations related to training load, cumulative fatigue, or adaptation status [37,38].

Countermovement jump assessment can be conducted with tools that trade off portability and breadth of metrics. Contact mats are highly portable and easy to use in the field but only provide limited variables (infer flight time to estimate jump height) [10,30]. In contrast, force platforms (e.g., AMTI) are considered the gold standard, offering greater reliability and a richer set of outcomes—including ground reaction forces—enabling precise quantification of key variables [32]. More recently, validated video-based smartphone applications such as MyJump2 leverage high–frame rate recording to estimate jump height with reasonable accuracy, expanding practical options for countermovement jump evaluation outside the laboratory [10,20,30]. Standardized technique instructions (e.g., hands on hips, no arm swing) remain essential across devices to ensure test–retest reliability [9,10,14]. Practitioners can evaluate both the magnitude and recovery trajectory of neuromuscular fatigue, facilitating more targeted adjustments to training and recovery strategies [14]. These portable solutions expand the feasibility of neuromuscular monitoring in field-based settings, offering practitioners a reliable way to track fatigue and readiness in functional fitness environments [32].

**Figure 2:**
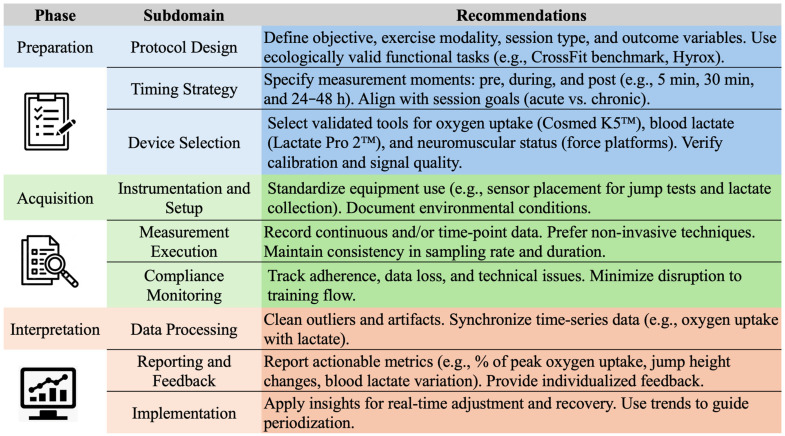
Recommendations on key elements of integrated physiological modelling for assessment of functional training and related exercise activities.

Beyond technological innovations, it is essential to establish clear and structured guidelines for the practical application of these tools in physiological monitoring. This work requires a systematic approach encompassing the definition of standardized protocols, appropriate timing for measurements, selection and calibration of validated instruments, criteria for data analysis, and recommended formats for reporting results. Such recommendations are critical to ensure the quality, consistency, and replicability of the data generated, both in research settings and everyday sports practice. Figure 2 provides a visual summary of these key elements, organized into the phases of preparation, acquisition, and interpretation. This operational model supports methodological standardization and effective implementation of physiological monitoring strategies in functional fitness environments.

Although advanced technologies like metabolic analyzers, lactate monitors, and force platforms offer valuable insights, their routine use in daily coaching is often limited by cost, accessibility, and required technical expertise. To enhance practical applicability, we recommend feasible and low-cost alternatives that align with the same physiological principles. Coaches can monitor training responses using accessible tools (e.g., heart rate monitors to track internal load, session rating of perceived exertion to estimate exertion) [13] and validated smartphone applications (like MyJump2 to assess neuromuscular fatigue) [20]. These methods provide sufficient resolution for day-to-day decisions in applied settings. Their use promotes individualization and responsiveness without relying on complex laboratory equipment.

## 4. Future Directions

A key unresolved question is how specific functional fitness structures (varying in duration, modality, and loading) differentially impact physiological and bioenergetic responses. To validate and expand the proposed physiological monitoring model, future research should conduct cross-sectional studies examining how different functional fitness structures elicit distinct acute physiological and bioenergetic responses. These sessions may vary in duration (e.g., short < 10 min vs. long > 20 min), modality (strength-based, metabolic conditioning, or mixed-modal), and loading strategy (fixed load vs. load relative to body mass). Including structured events such as Hyrox would provide an ecologically valid comparison to more variable CrossFit-style workouts. Such a design would allow researchers to explore how session characteristics influence variables such as oxygen uptake kinetics, post-exercise blood lactate concentration, heart rate and heart rate variability, neuromuscular fatigue, and underlying bioenergetic demands. This approach enables a more precise understanding of how different workout formats tax aerobic and anaerobic systems. Testing should be conducted in both male and female participants across a range of training experience (novice, intermediate, and advanced), with data collection at baseline, immediately post-exercise, and 24–72 h post-session to capture both acute responses and early recovery dynamics. Multiple regression models could then be applied to determine which markers best explain interindividual variability in performance, and whether this predictive capacity differs by sex, experience level, or workout type.

**Figure 3:**
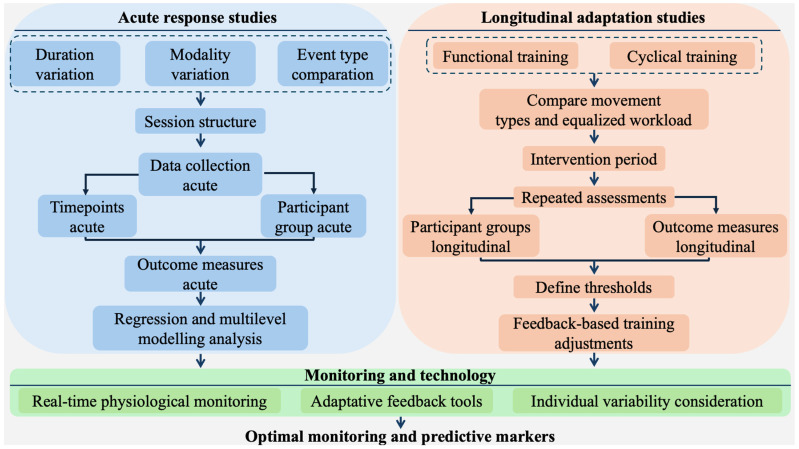
Methodological framework for investigating physiological demands in functional fitness, contrasting acute and longitudinal studies. The framework illustrates how different designs address short-term responses or long-term adaptations, and how monitoring technologies contribute to identifying optimal markers for training, recovery, and performance.

A relevant longitudinal research pathway involves examining whether different movement types, when performed under equalized external loads (volume, intensity, and density), lead to distinct adaptations. This approach would compare cyclical training formats (e.g., running and rowing) with functional multimodal protocols (e.g., Olympic lifts, burpees, and wall balls), maintaining identical workload parameters across conditions. An 8–12-week intervention with repeated assessments would allow researchers to analyze how movement structure influences physiological adaptation over time. Crucially, participants should include both males and females across varying levels of training experience (novice, intermediate, and advanced), to determine whether sex and training status modulate the response to different exercise formats. To operationalize this model, future studies should define actionable thresholds for each physiological marker (e.g., oxygen uptake amplitude recovery < 10% = adaptive; countermovement jump suppression > 8% at 48 h = high fatigue). Subsequent trials should test whether training adjustments based on these feedback signals improve practical outcomes, such as performance readiness, program adherence, or mitigation of non-functional overreaching and injury risk. Figure 3 details methodological pathways for investigating physiological demands in functional fitness, encompassing both acute response studies and longitudinal adaptation research.

## 5. Practical Applications

Real-time monitoring of oxygen uptake kinetics during functional fitness sessions allows coaches and practitioners to track aerobic system responses dynamically, enabling adjustments during sessions rather than relying solely on post-session analysis. Repeated assessments over time can also help quantify improvements in oxidative capacity or early signs of maladaptation, particularly when oxygen uptake amplitude or time constants show consistent trends across training blocks.

Combining neuromuscular fatigue diagnostics with individualized energy system profiling and adaptive feedback tools informs training prescription and load monitoring. Monitoring longitudinal trends (like persistent countermovement jump suppression, elevated heart rate variability variation, or disproportionate lactate accumulation under stable loads), offers insights into chronic training effects.

Portable technologies such as wearable oxygen analyzers, handheld lactate sensors, and compact force platforms facilitate accurate field-based assessments of internal load, expanding access to sophisticated physiological monitoring outside laboratory settings. Standardized protocols are required for data collection and interpretation (e.g., breath editing for oxygen uptake, consistent timepoints for lactate), along with consideration of individual variability based on sex, training status, and session type.

## 6. Conclusions

Integration of oxygen uptake kinetics, metabolic profiling, and neuromuscular fatigue diagnostics offers an effective framework for monitoring the internal demands of functional fitness. This approach captures physiological complexity often overlooked by conventional metrics, enabling better understanding of how athletes tolerate and adapt to fluctuating workloads. Using accessible non-invasive tools and real-time assessments, the model supports individualized decision-making grounded in physiological data. Practitioners can better align training and recovery with specific metabolic and fatigue profiles, optimizing performance while minimizing risk. Advanced wearable technologies and adaptive feedback systems enhance precision, expanding applicability beyond laboratories into everyday coaching. This model supports personalized, data-driven programming while bridging the gap between physiological research and practical coaching implementation.

## Data Availability

No new data were created or analyzed in this study.
